# Liquid/liquid displacement in a vibrating capillary

**DOI:** 10.1098/rsta.2022.0090

**Published:** 2023-04-17

**Authors:** Anatoliy Vorobev, Sergei Prokopev, Tatyana Lyubimova

**Affiliations:** ^1^ Faculty of Engineering and Physical Sciences, University of Southampton, Southampton SO17 1BJ, UK; ^2^ CFD Laboratory, Institute of Continuous Media Mechanics, Perm 614013, Russia; ^3^ Theoretical Physics Department, Perm State University, Perm 614990, Russia

**Keywords:** multiphase flow, phase-field modelling, vibrations, time-averaged approach, liquid/liquid displacement, capillary pressure

## Abstract

Mechanical vibrations can alter static and dynamic distributions of fluids in porous matrices. A popular theory that explains non-destructive changes in fluids percolation induced by vibrations involves elasticity of a solid matrix and compressibility of fluids. Owing to strong damping, elastic and acoustic deformations always remain bounded to narrow zones (a few centimetres) near the source of vibrations. However, field trials prove the existence of the effects that are induced by vibrations in geological reservoirs on a longer scale (100 m). In this study, we develop a non-elastic theory, assessing the time-averaged effects induced by small-amplitude high-frequency vibrations. We examine the immiscible liquid/liquid displacement flows in a capillary (which is a building element of a porous matrix) subjected to translational vibrations. We find that strong-enough vibrations alter the shapes of menisci and change the rates of displacement flows. We find that vibrations slow down or even stop the displacement flows (which is contrary to a common expectation that vibrations help to release fluids from a porous matrix).

This article is part of the theme issue ‘New trends in pattern formation and nonlinear dynamics of extended systems’.

## 1. Introduction

Dynamics of a liquid/liquid displacement in a porous medium is strongly determined by capillary forces. Owing to capillary action, displacement of a wetting phase is only possible when the pressure difference imposed between the ends of a porous sample overpowers the capillary pressure. The same physics underlies the effect of capillary trapping, when, for example, a sponge immersed in water and then taken out remains water saturated (water, despite being heavier than air, remains entrapped in a porous matrix).

The effect of capillary trapping is widely exploited by nature and industry for storage of liquids in porous media. For instance, oil production rates from mature oil fields are lower due to dispersion of oil phase into smaller blobs that are also immobilized by capillary forces. Obviously, there is a strong need for control and management of capillary forces. Heating and chemical additives are already widely employed for easier redistribution of liquids in a porous matrix. In this work, we aim to investigate if mechanical vibrations of a matrix can be an additional tool for effective (predictable) control of liquid/liquid displacements.

The action of capillary forces in a porous medium can be modelled by representing a porous medium by a network of capillaries (the so-called network approach). The capillary pressure in a porous matrix is then reduced to a multitude of capillary pressures associated with numerous interfaces that exist within elements of a matrix. In this work, we study the liquid/liquid displacement (movement of a liquid/liquid meniscus) in a single element of a porous matrix that is subjected to translational mechanical vibrations.

The application of oscillatory forcing for handling of multiphase fluid systems that saturate a porous medium is not a novel idea. Seismic stimulation of oil production from mature reservoirs was previously attempted in the Soviet Union in a series of field trials [1]. The idea was motivated by observations of the changes in water levels in wells and the changes in oil production noticed after passing trains or after remote earthquakes, i.e. that were induced by relatively weak seismic forcings. The idea was attractive for oil-producing industry due to the low cost, absence of harmful ecological consequences, and possibility of combining the new technique with conventional methods of enhanced oil recovery. However, the results of the trials were not always positive—only 40–50% of the trials brought improvements in oil production.

The inconsistency in the results was explained by insufficient understanding of the underlying physical mechanisms that explain the effect of vibrations on oil production. Through the further work, researchers identified more than a dozen physical mechanisms that are potentially capable of altering the flows in a porous medium. In particular, it was found that mechanical vibrations of very high-intensity (high amplitude and high frequency) rupture solid matrices producing mechanical destruction, improving local permeability in this way [2]. Ultrasonic vibrations (with wavelengths comparable with sizes of pores) may engage the effects of compressibility in either solid matrix or in fluids; these are peristaltic transport, when ultrasound deforms pore walls in the shape of travelling transversal waves [3], and the sonocapillary effect that is related to the acoustic cavitation [4]. Owing to high attenuation, ultrasonic vibrations work only locally, with maximum penetration lengths less than 10 cm. Ultrasound vibrations, in particular, are widely employed for facilitation of fluid flows in membranes and in other cleaning applications, which are also frequently modelled as porous media.

Despite the identification of a multitude of physical phenomena, a reliable, predictive and universal description of the action of vibrational forcing on fluids in a porous medium is still unavailable. Currently, the most popular theoretical explanation of the effect of vibrations on percolation of fluids is based on Biot’s theory that relates the acoustic force that pushes fluids through pores with elastic deformations of a solid matrix and fluids [5]. Again, as stated earlier, this theory is only applicable to the narrow zones near the vibration sources due to rapid decay of elastic deformations, so the effect is virtually non-existent at distances of a few centimetres. In field studies [1,6], however, there are observations that vibrations of lower frequencies (when effects of elasticity and compressibility do not play any role) are still capable of enhancing fluid flows, and such vibrations are capable of enhancing the flows on a larger spatial scale.

In this work, we aim to examine the effects of vibrations of lower frequencies. We develop a non-elastic theory that neglects elastic deformations of a solid matrix and fluids on a pore scale. We assume that the whole matrix oscillates with a small constant amplitude.

The period of vibrations that we examine is assumed to be much lower than any hydrodynamic time scale. An efficient description of such vibrations is achieved by the averaging approach, when the full governing equations are split into the two sets for separate descriptions of the pulsation and averaged flow fields. The primary focus of our study is on the averaged dynamics.

In particular, in heterogeneous fluid systems, high-frequency vibrations tend to induce steady (independent of short vibration time scale) deformation of interfaces, turning interfaces perpendicular to the direction of the vibrational forcing [7,8]. Application of this effect for altering the shapes of menisci and, potentially, for altering the dynamics of the liquid/liquid displacement has not yet been studied. This effect cannot be explained by Biot’s theory. A novel theory is needed, which we aim to develop in this work.

## 2. Physical model

### (a) Physical assumptions

We examine the dynamics of two immiscible and incompressible fluids that saturate a single capillary (a building element of a porous matrix) that is subjected to translational vibrations. We develop a new model [9] that describes the time-averaged effects of high-frequency vibrations on the behaviour of multiphase fluid systems. The model is based on the phase-field approach for the description of a heterogeneous binary mixture, and it can be equally used for the description of miscible and immiscible mixtures, by selection of the governing parameters. In this particular work, we focus on the immiscible interfaces, choosing the parameters that make the diffusive smearing of interfaces insignificant for the considered displacement runs. The model was previously validated by simulation of frozen waves induced by vibrations on an immiscible liquid/liquid interface [10].

The model presumes that vibrations are characterized by small amplitudes, ϕa≪h, and higher frequencies, $\omega \gg \nu /h^{2}$. Here, a and ω are the amplitude and circular frequency of vibrations, respectively, ϕ=Δρ/ρ is the density contrast, ν is the kinematic viscosity and h is the typical length (the diameter of a capillary). All flow variables are represented by a series using a small parameter (the ratio of the period of vibrations over the hydrodynamic time scale), and the analysis focuses on the leading orders of the governing equations. It is also assumed that all flow fields can be represented by small-amplitude pulsations induced by vibrations that develop on the slowly varying background. For instance, the fluid velocity is split into the pulsation and average components that are denoted by w and u, respectively. Furthermore, in the following equations, there are two orders of pulsation velocities, $w_{0}$ and w, which are the zeroth and first orders of this variable, respectively. The model presumes that the fluid mixture saturates a closed container, so that in the zeroth order, the fluid moves together with the container, as a solid body, and hence, $w_{0}$ is a constant vector. Inhomogeneities in the pulsational velocity induced by inhomogeneities in the field of concentration appear in the next order. These inhomogeneities are defined by w.

On the basis of the averaging approach, the system of equations can be split into separate descriptions of pulsation and average flows. The equations for the pulsation field are linear and inviscid. The equations for the average fields resemble the Boussinesq approximation of the Cahn–Hilliard–Navier–Stokes equations [11] with an added vibrational force.

We assume that the capillary is initially saturated with one liquid, and the other liquid is injected into the capillary from an inlet end; this injection results in displacement of the first liquid from the other (outlet) end. The capillary is subjected to translational vibrations. In this work, we consider two cases when the vector of vibrations is parallel and perpendicular to the capillary’s axis.

It is important to discuss the settings of the problem in more details. For the transversal vibrations (when the axis of vibrations and the axis of a capillary are perpendicular), the vibrations of the capillary will make an incompressible fluid move like a solid body together with the capillary. For the longitudinal vibrations, however, when the vibrations occur along the capillary’s centreline, the action of vibrations on the fluid that saturate a capillary could be different. Since the pulsation motion is essentially inviscid, in a straight capillary with two open ends, the fluid would simply slip along the walls, and neither the pulsation nor average motion could be induced in the bulk region, at least to first order in the expansion of the governing equations. We do not consider this case.

**Figure 1.**
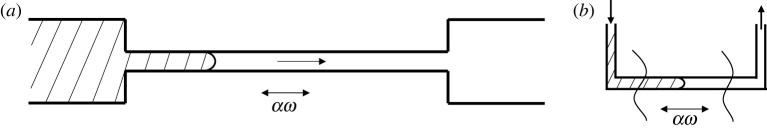
(*a*) A capillary joining two pores. The whole matrix is subjected to translational vibrations. (*b*) A capillary of a complex shape.

In our work, we assume that the capillary is a part of a larger matrix (the capillary joins two pores of a porous matrix like that shown in figure 1*a*, or the capillary may have a complex shape, as shown in figure 1*b*). We do not consider the other elements of the matrix (and we only consider the straight part of a complex capillary), but we assume that the whole matrix is subjected to vibrations and that the fluids that saturate the matrix fulfil oscillatory movements together with the solid matrix (to leading order the fluid that saturates a matrix moves like a solid body together with the solid matrix). In this work, we will focus on the effect of vibrations induced in a single capillary, far from any boundaries that are perpendicular to the capillary’s centreline, but we assume that such boundaries do exist. These boundaries will make the fluid vibrate together with the walls. The consideration of the pulsation field near these boundaries is outside the scope of the current work.^1^

### (b) Governing equations

The governing equations for the averaged flow fields read,

2.1 $$\begin{array}{rl} & \frac{\partial u}{\partial t}+(u\cdot \nabla )u=-\nabla \frac{\Pi }{\rho }+\frac{\eta }{\rho }\nabla^{2}u-C\nabla \mu -\frac{\phi }{2}(w\cdot w_{0})\nabla C, \end{array}$$

2.2 $$\begin{array}{rl} & \frac{\partial C}{\partial t}+(u\cdot \nabla )C=\frac{\alpha }{\rho }\nabla^{2}\mu , \end{array}$$

2.3 $$\begin{array}{rl} & \nabla \cdot u=0, \end{array}$$

2.4 $$\begin{array}{rl} & \mu =-\phi (g\cdot r)+2a_{T}C+4bC^{3}-\epsilon \nabla^{2}C \end{array}$$

2.5 $$\begin{array}{rl} \mathrm{and}\; & \Pi =p+\rho \left(\frac{w^{2}}{4}+\epsilon \frac{{\left(\nabla C\right)}^{2}}{2}-\mu C+a_{T}C^{2}+bC^{4}-\phi (g\cdot r)C\right). \end{array}$$

Here, C is the concentration, defined as the mass fraction of one of the liquids in a mixture, μ is the chemical potential (in the phase-field approach, the diffusion flux is proportional to the gradient of the chemical potential, and intensity of diffusion is controlled by the mobility coefficient α, which is proportional to the standard coefficient of diffusion), the coefficients $a_{T}$ and b are the two parameters that define the thermodynamic model of the binary mixture, p is the pressure and Π is the modified pressure (that includes all gradient terms in the Navier–Stokes equation). The Navier–Stokes equation includes the Korteweg force that determines the morphology of the liquid/liquid boundary, and the vibrational force. The gravity term enters the definition of the chemical potential (this term will also enter the Navier–Stokes equation if the chemical potential is substituted into the Korteweg force). The gravity effect is determined by the gravitational acceleration g and by the density contrast ϕ.

The vector w determines the spatial variations in the pulsational velocity, and it is defined by equations:

2.6 $$\nabla \times w=\phi w_{0}\times \nabla C;\;\nabla \cdot w=0.$$

To non-dimensionalize equations (2.1)–(2.6), we use the following scales for the length, time, average velocity, pulsational velocity, pressure and chemical potential:

2.7 $$h,\;\frac{h}{V_{*}},\;V_{*}=\frac{\Delta p}{L}\frac{h^{2}}{8\eta_{*}},\;\phi a\omega ,\;\rho V_{*}^{2},\;\mu_{*}=b.$$

Here, h and L are the diameter and length of a capillary, respectively. The typical velocity, $V_{*}$, is defined as the maximum velocity of the Poiseuille’s flow (that would exist in a capillary subjected to the pressure difference $\Delta p=p_{1}-p_{2}$ between the ends if the surface tension effects are ignored).

Also, we redefine the zeroth order of the pulsational velocity as $w_{0}\to a\omega w_{0}$, with $w_{0}$ becoming a dimensionless unit vector that defines the direction of vibrations. Similarly, the vector of gravity acceleration is redefined as g→−gγ, where g is the magnitude of the gravity acceleration and γ is the unit vector that is directed upwards.

The dimensionless form of equations (2.1)–(2.6) reads,

2.8 $$\begin{array}{rl} & \frac{\partial u}{\partial t}+(u\cdot \nabla )u=-\nabla \Pi +\frac{1}{Re}\nabla^{2}u-\frac{1}{M}C\nabla \mu -V(w\cdot w_{0})\nabla C, \end{array}$$

2.9 $$\begin{array}{rl} & \frac{\partial C}{\partial t}+(u\cdot \nabla )C=\frac{1}{Pe}\nabla^{2}\mu , \end{array}$$

2.10 $$\begin{array}{rl} & \nabla \cdot u=0, \end{array}$$

2.11 $$\begin{array}{rl} & \nabla \times w=w_{0}\times \nabla C;\;\nabla \cdot w=0, \end{array}$$

2.12 $$\begin{array}{rl} & \mu =\frac{Gr}{M}(\gamma \cdot r)+2AC+4C^{3}-Cn\nabla^{2}C \end{array}$$

2.13 $$\begin{array}{rl} \mathrm{and}\; & \Pi =p+V\frac{w^{2}}{2}+\frac{Cn}{M}\frac{{\left(\nabla C\right)}^{2}}{2}-\frac{1}{M}(\mu C-AC^{2}-C^{4})-Gr(\gamma \cdot r)C. \end{array}$$

Equations (2.8)–(2.13) include the following non-dimensional parameters,

2.14 $$Re=\frac{\rho_{*}hV_{*}}{\eta_{*}}\;\mathrm{and}\;Pe=\frac{\rho_{*}hV_{*}}{\alpha \mu_{*}}.$$

These are the Reynolds and Peclet numbers that determine the roles of the viscous and diffusive effects. In the phase-field approach, the diffusivity can be defined as $D_{*}=(\alpha \mu_{*}/\rho_{*})$. The next two non-dimensional parameters are defined as follows:

2.15 $$Gr=\frac{\phi gh}{V_{*}^{2}}\;\mathrm{and}\;V=\frac{{\left(\phi a\omega \right)}^{2}}{2V_{*}^{2}}.$$

The Grashof number determines the role of the gravity effect. The vibrational parameter V, i.e. proportional to the square of the vibrational velocity, defines the strength of the vibrational force. The two other non-dimensional parameters are as follows:

2.16 $$M=\frac{V_{*}^{2}}{\mu_{*}}\;\mathrm{and}\;Cn=\frac{\epsilon }{\mu_{*}h_{*}^{2}}.$$

The Mach number (that should not be confused with the classical Mach number) determines the strength of the Korteweg force and hence determines the strength of the surface tension forces. The Cahn number controls the thickness of the interface (i.e. proportional to $\sqrt{-Cn/A}$). Finally, the parameter

2.17 $$A=\frac{a_{T}}{b}$$

determines the thermodynamic behaviour of a binary mixture.

In this work, we focus on immiscible interfaces, which we achieve by considering relatively short capillaries and relatively high Peclet numbers and also by taking A=−1/2 (i.e. taking the temperature of a binary mixture below the critical point).

The liquid/liquid displacement is strongly determined by the capillary effects. The strength of the capillary effects is traditionally determined by the surface tension coefficient. Within the phase-field approach, the surface tension forces are determined by a number of alternative parameters, while the surface tension coefficient can be calculated from the following equation:

2.18 $$\sigma =\frac{Re\;Ca}{M}\frac{E_{i}}{S}=\frac{Re\;Ca\;Cn}{M}\frac{1}{S}\int_{V}{\left(\nabla C\right)}^{2}\;dV.$$

Here, $E_{i}$ is the interfacial energy that is scaled by $\rho \mu_{*}h_{*}^{3}$, σ is the coefficient of surface tension that is scaled by $\sigma_{*}$ (the dimensional value of the surface tension coefficient) and S is the area of the interface scaled by $h_{*}^{2}$. The latest equation also introduces one more non-dimensional parameter, the capillary number,

2.19 $$Ca=\frac{\eta_{*}V_{*}}{\sigma_{*}}.$$

### (c) Values of non-dimensional parameters

To estimate the values of the non-dimensional parameters, we assume that we examine the evolution of an oil/water mixture (with typical density $\rho_{*}∼10^{3}\;\mathrm{kg}\;m^{-3}$, density contrast ϕ∼0.1, viscosity $\eta_{*}∼10^{-3}\;\mathrm{Pa}\;s^{-1}$, diffusivity $D_{*}∼10^{-9}\;m^{2}\;s^{-1}$ and surface tension $\sigma_{*}∼0.01\;N\;m^{-1}$) that saturates a capillary (with radius $h∼10^{-4}\;m$ and length $L∼10^{-3}\;m$). The flow in a capillary is driven by the pressure difference, Δp∼1 Pa (this could be derived from the assumption that the overall pressure gradient is $10^{4}\;\mathrm{Pa}\;m^{-1}$), with the typical velocity $V_{*}∼10^{-3}\;m\;s^{-1}$.

These assumptions allow us to conclude that $Ca∼10^{-4}$, Re∼0.1, Pe=100, Gr∼100. Here we assume that the gravity is directed perpendicular to the capillary axis. For a vertical capillary, the value of the Grashof number would be greater.

The capillary is subjected to the vibrations. We assume that elastic deformations of a solid matrix and fluids are negligible. Indeed, deformation of a porous matrix may only be important on a length scale that is comparable with the wavelength of the waves induced by vibrations, λ∼c/ω, where c is the speed of sound ($c∼10^{3}\;m\;s^{-1}$) and ω is the frequency of vibrations. For low-frequency vibrations, the wavelength can be as high as 1 km, and even for ultrasonic vibrations, λ is greater than 1 cm. Our focus is on the capillary effects that are determined by the shape of a liquid/liquid meniscus in a single pore, and hence, the deformation of a porous matrix remains unimportant for our study. We also assume that vibrations are non-acoustical, again, in the sense that the wavelength of induced waves is considerably greater than the capillary’s diameter, ω≪c/h. For non-acoustical vibrations, compressibility effects may be neglected even for pulsation motion.

At the same time, the period of vibrations is assumed to be considerably smaller than the typical convective and diffusive time scales (that are calculated for the size of a single pore), namely,

2.20 $$\frac{1}{\omega }\frac{\eta_{*}}{\rho_{*}h^{2}}\ll 1\;\mathrm{and}\;\frac{1}{\omega }\frac{D_{*}}{h^{2}}\ll 1,$$

or, in terms of non-dimensional parameters,

2.21 $$\frac{1}{V^{1/2}Re}\frac{\phi a}{h}\ll 1\;\mathrm{and}\;\frac{1}{V^{1/2}Pe}\frac{\phi a}{h}\ll 1.$$

Let us assume that the amplitude and frequency of vibrations are $a∼10^{-4}\;m$ and $\omega ∼10^{3}\;s^{-1}$, respectively, which makes V∼100. These parameters, however, especially frequency, can be easily changed which can be reflected by the change of the vibrational parameter V. For the considered level of vibrations, the typical value of the pulsation velocity is $0.1\;m\;s^{-1}$.

The value of the Mach number determines the strength of the capillary force. The definition of this parameter involves non-standard phenomenological parameters, which makes its estimation difficult. In our previous works, we noted that the value of the Mach number affects a few different effects, including the shape of the interface and the sliding speed of the contact line [12]. In this work, we fulfil calculations for different Mach numbers, examining the cases of lower capillary forces (the fingering displacement) and higher capillary forces (when the meniscus is compact, symmetrical, and is moving like a piston).

The value of the Cahn number determines the interface thickness. The real interface thickness is very small, a few molecular layers, which is zero for the macroscopic theory that is developed in this work. For the sake of numerical calculations, however, the interface is smeared, represented by a boundary of finite thickness. To reproduce the physically relevant behaviour, the Cahn number needs to be taken very small, which makes the numerical simulations challenging due to the need to resolve a small interface thickness. We take the Cahn number so small that the interface thickness becomes the smallest size that characterizes the problem, much smaller than any other size, which is equivalent to considering the limit of zero Cahn number.

## 3. Problem statement

In this work, we consider a two-dimensional geometry, representing the capillary by a rectangular domain. The coordinates along and across the capillary are denoted by x and y, respectively. We examine the action of the translational vibrations that are directed either parallel or perpendicular to the capillary’s axis, naming them the longitudinal ($w_{0}=(1,0)$) and transversal ($w_{0}=(0,1)$) vibrations, respectively. Also, we consider the different orientations of the capillary in a gravity field, the horizontal (γ=(1,0)) and vertical (γ=(0,1)) capillaries.

To avoid difficulties in the formulation of the boundary conditions at the inlet and outlet ends, at t=0, we place the meniscus inside the capillary, at the position x=0.5. The meniscus separates two liquids with concentration levels C=−1/2 (displacing liquid) and C=1/2 (displaced liquid). The pressure difference that induces the flow is switched on at time t=0.

Our analysis is restricted to the moments when a meniscus stays sufficiently far away from the capillary’s inlet and outlet ends. We fulfilled the calculations for capillaries of different lengths, from $L_{x}=10$ to $L_{x}=50$; however, the results reported below are obtained for $L_{x}=10$, when we already obtain a sufficiently long dynamics unaffected by the capillary’s ends.

The governing equations are supplemented with the boundary conditions. The motion on a long time scale (the average motion) is primarily set by the pressure difference that is imposed between the ends of the capillary. The reference point for the pressure field is set at the right (outlet) end ($p_{2}=0$). The pressure level at the left (inlet) end of a capillary is always set as $p_{1}=8L/Re$. In a single-phase flow, this pressure difference, $(p_{1}-p_{2})=8L/Re$, will generate the Poiseuille’s flow with the flow rate Q=2/3. The actual flow rate will in fact be lower due to the action of capillary forces.

At the walls, for the average velocity, we set the no-slip boundary condition (u=0), the absence of the diffusion flux through the wall (∂μ/(∂y)=0) for the chemical potential and the condition for the concentration field that defines the condition of neutral wetting (the contact line is orthogonal to the wall, the molecules of both liquids interact equally with the molecules of the wall), which is defined by ∂C/(∂y)=0.^2^

We need a separate discussion of the boundary conditions for the pulsation velocity w. It is easy to note that the differential equations for the pulsation velocity (2.11) are of lower order (compared with the equations for the average flow), and hence, these equations require fewer boundary conditions. For instance, at the walls, we may impose the no-penetration condition, but we cannot impose the no-slip condition (as the pulsation flow is inviscid).

To solve the pulsational problem, it is convenient to introduce the potential of the pulsational velocity, Φ,

3.1 $$w=-Cw_{0}+\nabla \Phi .$$

Then, in terms of the velocity potential, the problem for the pulsational flow reads,

3.2 $$\nabla^{2}\Phi =\nabla C\cdot w_{0}.$$

Recall that we describe the action of vibrations by using a non-inertial reference frame that is attached to the vibrating capillary, and hence in terms of velocity, the no-penetration boundary condition at the walls is $w_{y}=0$. In terms of the velocity potential, the no-penetration condition is ∂Φ/∂y=0 for the longitudinal vibrations and ∂Φ/∂y=C for the transverse vibrations.

At the inlet/outlet ends of the capillary, the boundary conditions for the pulsational flow are chosen so as to minimize the influence of the ends on the flow fields. The analysis of equation (3.2) shows that the velocity potential must be either constant or a linear function of the coordinates far from interfaces (where the concentration field is homogeneous). In addition, the velocity potential is defined with the accuracy of an arbitrary constant. Taking into account these constraints, at the inlet end of the capillary (at x=0) for the longitudinal vibrations, we simply set Φ=0, and for the transversal vibrations, we set Φ=y/2. At the outlet end, at $x=L_{x}$, we set a ‘soft’ condition, $\partial^{2}\Phi /\partial x^{2}=0$ (we presume that the pulsational flow is fully established at this end, or, in other words, the x-component of the pulsation velocity does not change along the capillary).

The governing equations are solved numerically using the formulation of the primitive variables, pressure–velocity, and using the fractional-step (or projection) method that is implemented on the basis of the finite difference approach [12]. The Poisson equations for the pressure and for the potential of the pulsation velocity are solved by using the iterative Gauss–Seidel method. We developed a novel algorithm for effective solution of the equations with the use of graphical processing units. The necessity to trace the interface between two liquids enforces higher requirements on the numerical resolution. We conduct the calculations using a uniform mesh with the size of 1/400 across and along the capillary. In our earlier works [12,13,15], we found that this resolution is sufficient for the accurate reproduction of the dynamics of a two-phase fluid mixture in a capillary (still, we reproduced some of our calculations using the resolutions 1/300 and 1/500, and found minor, almost non-existent, differences in the results).

It is also important to note what happens if a longer capillary is taken for calculations. In this case, the pressure boundary condition makes the pressure gradient along the capillary stronger, but the value of the capillary pressure remains unchanged. By keeping all parameters unchanged and by simply varying the capillary’s length, one would alter the role of capillary forces and thus change the flow rate. To keep the dynamics unaltered, changes in the capillary’s length should be correlated with the changes of the Mach number. Similarly, changes in the Cahn and Mach numbers should also be coordinated [12,13].

## 4. Numerical results

### (a) Basic flow no vibrations

First, we would like to review the basic features of the liquid/liquid displacement in a capillary that is not subjected to vibrational forcing. A brief summary of this basic case is still needed for interpretation of the effects of vibrations. Further results on the displacement flow with no vibrations can be also found in our recent publications [12,13,15].

**Figure 2.**
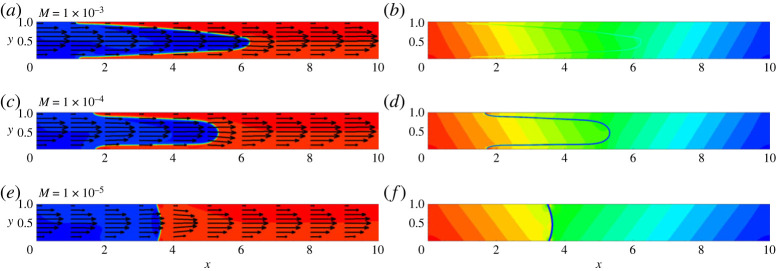
The fields of concentration and velocity (*a*,*c*,*e*) and the fields of pressure (*b*,*d*,*f*) at t=6. The snapshots are shown for a horizontal capillary with no vibrations applied. The calculations are fulfilled for the following parameters: A=−0.5, $Cn=10^{-4}$, Re=0.1, Pe=100, Gr=100, and for three different Mach numbers, $M=10^{-3}$ (*a*,*b*), $M=10^{-4}$ (*c*,*d*) and $M=10^{-5}$ (*e*,*f*). (Online version in colour.)

In this study, we focus on immiscible interfaces. A key parameter that defines the shape and propagation of menisci is the surface tension. For the case of lower surface tensions, the meniscus is continuously stretched, so the liquid that originally saturates the capillary is getting displaced only from the middle section (the fingering displacement). For the case of stronger surface tension forces, the interface remains compact, and the full (piston-like) displacement is observed. In the phase-field simulations, the surface tension is controlled by the Mach number, and the results for different Mach numbers are shown in figure 2. One can see that at higher Mach numbers, the role of the surface tension is negligible, so the fingering displacement occurs. At lower Mach numbers, however, the displacement front has a piston-like shape. For all Mach numbers, the flow has a parabolic velocity profile far from the meniscus. In case of lower Mach numbers, there is a narrow zone near the meniscus tip where the flow profile is different from its classical parabolic shape. In the pressure fields, one can also see a pressure jump across the meniscus (the capillary pressure). Also, inclination of the pressure isolines reflects the influence of the gravity force.

**Figure 3.**
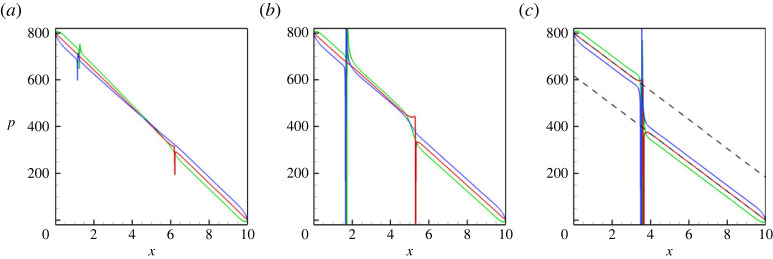
The pressure profiles along the capillary are shown for the three different positions: (green line) at the lower wall, y=0, (red line) at the centreline, y=1/2 and (blue line) at the upper wall, y=1. The profiles are shown for a horizontal capillary with no vibrations applied, at t=6. The calculations are fulfilled for the following parameters: A=−0.5, $Cn=10^{-4}$, Re=0.1, Pe=100, Gr=100, and three different Mach numbers, $M=10^{-3}$ (*a*), $M=10^{-4}$ (*b*) and $M=10^{-5}$ (*c*). In (*c*), the dashed lines depict the curves defined by the equations, $(p_{2}-p_{1})-(p_{2}-p_{1}-p_{c})x/L_{x}$ and $(p_{2}-p_{1}-p_{c})-(p_{2}-p_{1}-p_{c})x/L_{x}$, where $p_{1}=800$, $p_{2}=0$, $p_{c}=183$ and $L_{x}=10$. (Online version in colour.)

All aforementioned conclusions are also supported by the pressure profiles that are shown in figure 3. In particular, in figure 3, one understands that the value of the pressure drop along the capillary is imposed by the pressure boundary conditions, the pressure levels along the lower and upper walls are slightly different due to the effect of gravity, and the pressure experiences a jump across the meniscus, whose value is associated with the capillary pressure.

**Figure 4.**
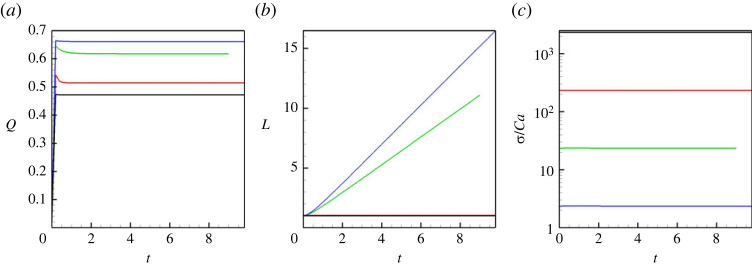
(*a*) The volumetric flow rate through the capillary versus time. (*b*) The length of a meniscus versus time. (*c*) The surface tension coefficient divided by the capillary number versus time. The results are shown for a horizontal capillary with no vibrations applied. The calculations are fulfilled for the following parameters: A=−0.5, $Cn=10^{-4}$, Re=0.1, Pe=100, Gr=100, and four different Mach numbers, $M=10^{-3}$ (blue line), $M=10^{-4}$ (green line), $M=10^{-5}$ (red line) and $M=10^{-6}$ (black line). (Online version in colour.)

We also show a few integral characteristics that define the liquid/liquid displacement. In particular, figure 4*a* depicts the volumetric flow rate. At higher Mach numbers, the capillary pressure is low, so the flow rate in the capillary is very close to 2/3, which is the value for the classical Poisseuile flow for a single-phase fluid. An increase in the capillary forces slows down the displacement flow. Figure 4*b* depicts the length of the meniscus: again, one can see that at higher Mach numbers menisci are constantly stretched, and at lower Mach numbers, the length of the meniscus remains constant, close to 1, which is the value of the capillary’s diameter. Finally, figure 4*c* shows the values of the surface tension (that are divided by the capillary number), which are $2.38\times 10^{-4}$ for $M=10^{-3}$, $2.37\times 10^{-3}$ for $M=10^{-4}$, $2.34\times 10^{-2}$ for $M=10^{-5}$ and 0.234 for $M=10^{-6}$ (these values are given for the capillary number $Ca=10^{-4}$).

We should also note that there is a simple relationship between the capillary pressure and the flow rate, Q [12]:

4.1 $$\frac{p_{c}}{\left(p_{1}-p_{2}\right)}=\left(1-\frac{Q}{Q_{1}}\right).$$

Here, $Q_{1}=2/3$ is the volumetric flow rate for the single-phase flow. This relationship gives an easy way to calculate the capillary pressure, which is 6.8 for $M=10^{-3}$, 58.4 for $M=10^{-4}$, 183 for $M=10^{-5}$ and 234 for $M=10^{-6}$. In figure 3*c*, we also show that these values of the capillary pressure match the pressure profiles, correctly predicting the pressure jumps across the menisci.

**Figure 5.**
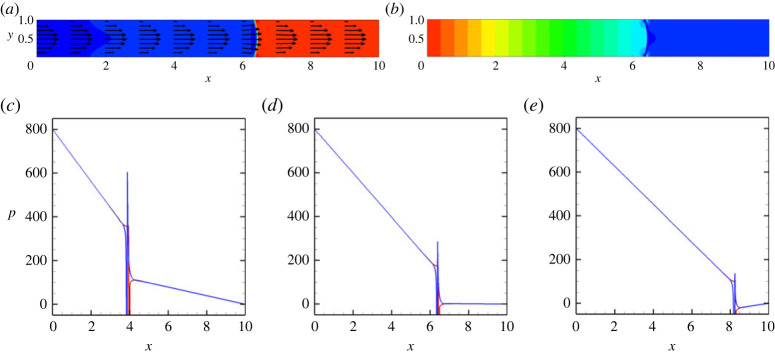
The fields of concentration and velocity (*a*) and the fields of pressure (*b*) at t=10 for a vertical capillary with no vibrations applied. (*c*–*e*) The pressure profiles along the capillary for the three different positions: (green line) at the lower wall, y=0, (red line) at the centreline, y=1/2 and (blue line) at the upper wall, y=1. The profiles are shown for t=5 (*c*), t=10 (*d*) and t=15 (*e*). The calculations are fulfilled for the following parameters: A=−0.5, $Cn=10^{-4}$, Re=0.1, Pe=100, Gr=100 and $M=10^{-5}$. (Online version in colour.)

**Figure 6.**
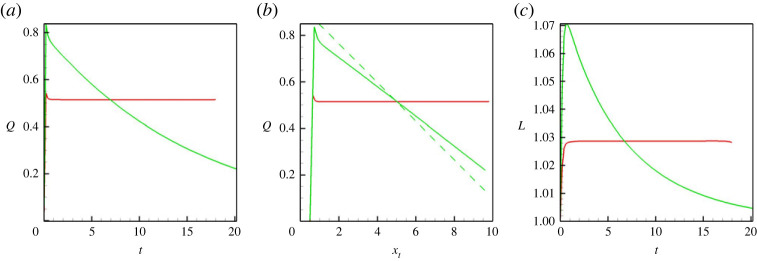
(*a*) The volumetric flow rate through the capillary versus time. (*b*) The volumetric flow rate through the capillary versus position of the tip of the meniscus. (*c*) The length of a meniscus versus time. The results are shown for the horizontal (red lines) and vertical (green lines) capillaries with no vibrations. The calculations are fulfilled for the following parameters: A=−0.5, $Cn=10^{-4}$, Re=0.1, Pe=100, Gr=100 and $M=10^{-5}$. In (*b*), the dashed line is defined by equation (4.2). (Online version in colour.)

Finally, we review the results for a vertical capillary. The corresponding flow fields are depicted in figure 5*a*,*b*. One can see that the flow has the parabolic velocity profile, and the pressure isolines are symmetric in respect to the capillary’s centreline. However, the distribution of the pressure along the capillary becomes more complex. The pressure distribution becomes a function of the position of a meniscus, see figure 5*c*–*e*, which is explained by the addition of the hydrostatic pressure, whose value can be approximated by $1/2GrL_{x}(1-2x_{t}/L_{x})$, where $x_{t}$ is the position of the meniscus tip. These observations are also confirmed in figure 6.

In figure 6*a*, it is easy to notice that the rate of the displacement flow is affected by the gravity force. The gravity force is proportional to the average concentration of the mixture that saturates a capillary (as density is proportional to concentration). At the initial moment, at t=0, the average concentration is 1/2, and at the later moments, the average concentration gets smaller. Consequently, the effect of the gravity is stronger at the first instances (when gravity assists the displacement), decreasing on progression of the second liquid into the capillary. When the meniscus moves through the middle of the capillary, the average concentration changes its sign, so the gravity force changes its sign as well, starting to slow down the displacement.^3^ Approximately, the effect of gravity in a vertical capillary can be defined by the following modified equation (4.1):

4.2 $$Q=Q_{1}\left(1-\frac{1}{p_{1}-p_{2}}\left(p_{c}-\frac{1}{2}GrL_{x}\left(1-2\frac{x_{t}}{L_{x}}\right)\right)\right).$$

Interpretation of the results for vertical capillaries, due to the position-dependent gravity effect, becomes confusing, and that is the reason why we present most of the results below for horizontal capillaries.

### (b) Transversal vibrations

**Figure 7.**
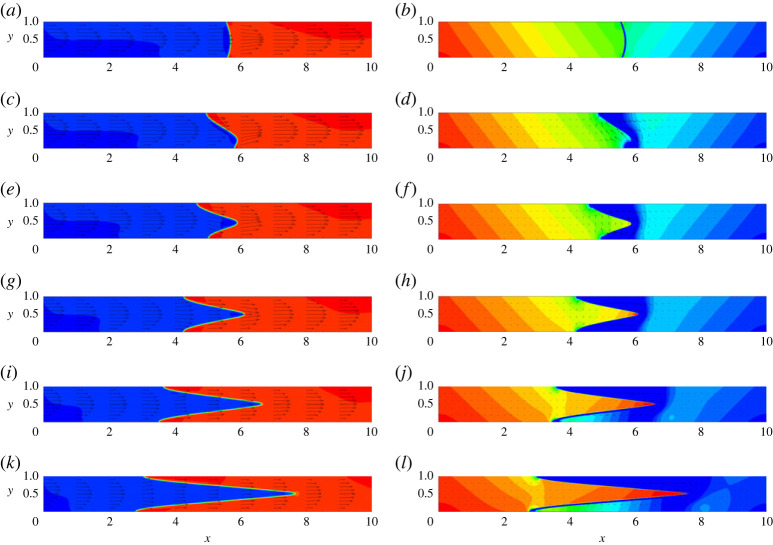
The fields of concentration and velocity (*a*,*c*,*e*,*g*,*i*,*k*) and the fields of pressure and pulsation velocity (*b*,*d*,*f*,*h*,*j*,*l*) at t=10. The snapshots are shown for the horizontal capillary that is subjected to transversal vibrations. The calculations are fulfilled for the following parameters: A=−0.5, $Cn=10^{-4}$, Re=0.1, Pe=100, Gr=100, $M=10^{-5}$ and for five levels of vibrations, V=0 (*a*,*b*), V=8000 (*c*,*d*), V=16 000 (*e*,*f*), V=32 000 (*g*,*h*), V=64 000 (*i*,*j*) and V=128 000 (*k*,*l*). (Online version in colour.)

Let us first examine the case when the capillary is vibrated in the direction perpendicular to the capillary’s axis. In figure 7, one can see the typical shapes of menisci in horizontal capillaries subjected to vibrations of different intensities. Only very strong vibrations, with the values of the vibrational parameter $V>10^{3}$, induce any noticeable (visible) effects on the displacement flow. Vibrations are stretching the meniscus, reshaping it, so the greater area of an interface becomes perpendicular to the direction of vibrations. In particular, at higher levels of vibrations, the meniscus becomes stretched along the capillary, taking the shape of a very sharp cone. For the average flow, the parabolic velocity profile can still be observed in the capillary (again, except for the narrow region near the meniscus). The pressure profile becomes different in front of the meniscus, where the low-pressure region can be observed. The pulsational flow is generated near the meniscus, and it has the shape of a clockwise vortex. In figure 7*d*,*f*,*h*, one may notice a weak pulsational flow at some considerable distance from the meniscus. We found that these smaller values of the pulsational velocity are eliminated by fulfilling calculations for longer capillaries.

**Figure 8.**
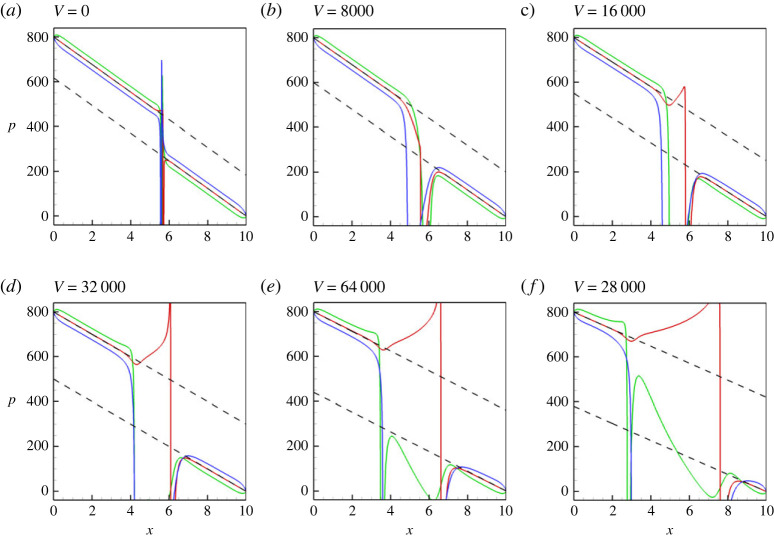
The pressure profiles along the capillary are shown for the three different positions: (green line) at the lower wall, y=0, (red line) at the centreline, y=1/2, and (blue line) at the upper wall, y=1. The profiles are shown for the horizontal capillary subjected to transversal vibrations at t=10. The calculations are fulfilled for the following parameters: A=−0.5, $Cn=10^{-4}$, Re=0.1, Pe=100, Gr=100, $M=10^{-5}$, and for different levels of vibration forcing (as indicated in the figure). The dashed lines are defined by the equations $(p_{1}-p_{2})-(p_{1}-p_{2}-p_{c})x/L$ and $(p_{1}-p_{2}-p_{c})-(p_{1}-p_{2}-p_{c})x/L$, where $p_{1}=800$, $p_{2}=0$, L=10 and $p_{c}=183$ for V=0, $p_{c}=200$ for V=8000, $p_{c}=250$ for V=16 000, $p_{c}=300$ for V=32 000, $p_{c}=360$ for V=64 000 and $p_{c}=420$ for V=128 000. (Online version in colour.)

**Figure 9.**
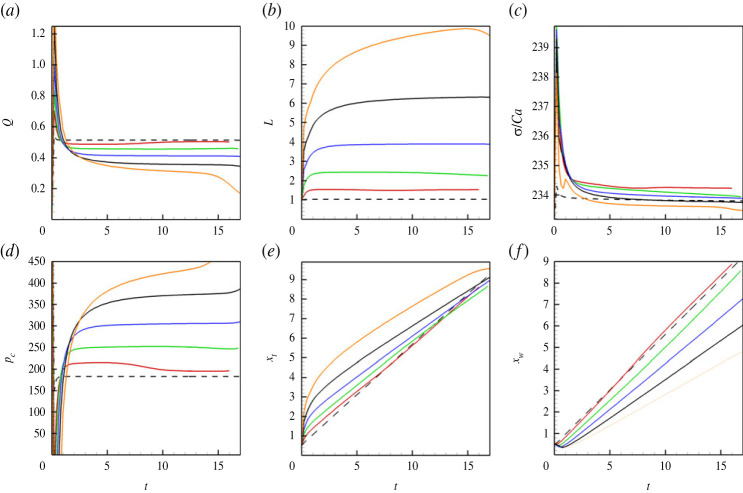
(*a*) The volumetric flow rate through the capillary versus time. (*b*) The length of a meniscus versus time. (*c*) The surface tension coefficient divided by the capillary number versus time. (*d*) The capillary pressure versus time. (*e*,*f*) The positions of the meniscus tip and of the contact line versus time. The results are shown for a horizontal capillary subjected to transversal vibrations. The calculations are fulfilled for the following parameters: A=−0.5, $Cn=10^{-4}$, Re=0.1, Pe=100, Gr=100, $M=10^{-5}$, and for different values of the vibrational parameter V: V=0 (dashed line), V=8000 (red line), V=16 000 (green line), V=32 000 (blue line), V=64 000 (black line) and V=128 000 (orange line). (Online version in colour.)

More details on the pressure profiles are shown in figure 8, where one notices that an increase in the level of vibrations is correlated with an increase of the capillary pressure (the pressure jump across an interface). Further, the change in the capillary pressure is associated with the change of the flow rate through the capillary, which can be seen in figure 9*a*. In figure 9*a*, one can see that stretching of a meniscus by vibrations (see also figure 9*b*) results in a rapid displacement (squeezing) of a liquid from the capillary. However, later, when the shape of a meniscus is already stabilized, the movement of the meniscus is slowed down by the capillary forces. The coefficient of the surface tension (figure 9*c*) remains almost independent of the level of vibrations (there is only a slight increase at the first instances, when stretching of a meniscus makes the interface thinner, which increases the surface tension coefficient [16]). The capillary pressure that is determined from equation (4.1) is depicted in figure 9*d*. Finally, figure 9*e*,*f* depict the speeds of the tip (in the centre) and of the ends (at the walls) of the menisci.

**Figure 10.**
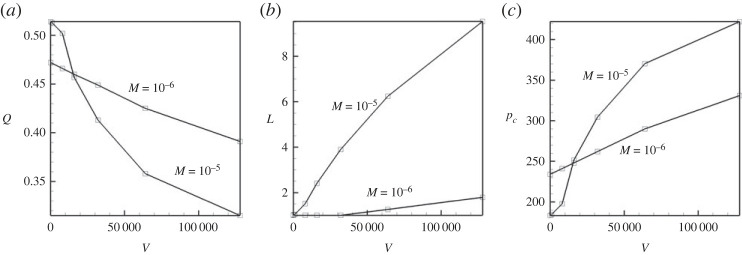
The volumetric flow rate (at long times) through the capillary (*a*), the length of the meniscus (*b*) and the capillary pressure (*c*) versus the vibrational parameter V. The results are shown for the horizontal capillary subjected to transversal vibrations. The calculations are fulfilled for the following parameters: A=−0.5, $Cn=10^{-4}$, Re=0.1, Pe=100, Gr=100, $M=10^{-5}$ and $M=10^{-6}$.

In figure 10, we summarize the effect of the transversal vibrations. Such vibrations deform the meniscus, stretching it along the capillary (figure 10*b*). The capillary pressure associated with the interface is also increased (figure 10*c*), which results in slowing down of the displacement flow (figure 10*a*). The effect of vibrations depends on the Mach number (surface tension), so for deformation of an interface with higher surface tension coefficient, a stronger level of vibrational forcing is needed.

We would like to note that the pressure in a capillary is determined by the shape of the interface, apparent contact angle and surface tension (see [13,15]). In this work, we show that vibrations are not able to change the surface tension coefficient (in other words, vibrations do not change the distributions of fluids within a thin interface), but vibrations can alter shapes of interfaces, thus altering the capillary pressures associated with interfaces, and altering the rates of displacement flows.

**Figure 11.**
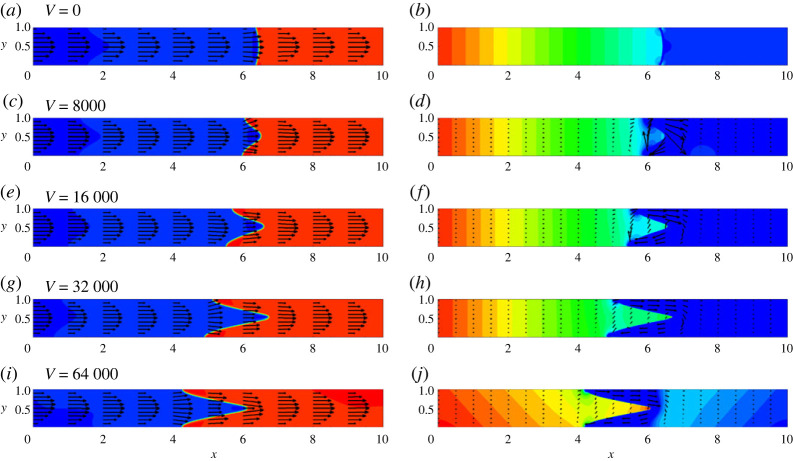
The fields of concentration and velocity (*a*,*c*,*e*,*g*,*i*) and the fields of pressure and pulsation velocity (*b*,*d*,*f*,*h*,*j*) at t=10. The snapshots are shown for a vertical capillary that is subjected to transversal vibrations. The calculations are fulfilled for the following parameters: A=−0.5, $Cn=10^{-4}$, Re=0.1, Pe=100, Gr=100, $M=10^{-5}$ and for five levels of vibrations, V=0 (*a*,*b*), V=8000 (*c*,*d*), V=16 000 (*e*,*f*), V=32 000 (*g*,*h*) and V=64 000 (*i*,*j*). (Online version in colour.)

**Figure 12.**
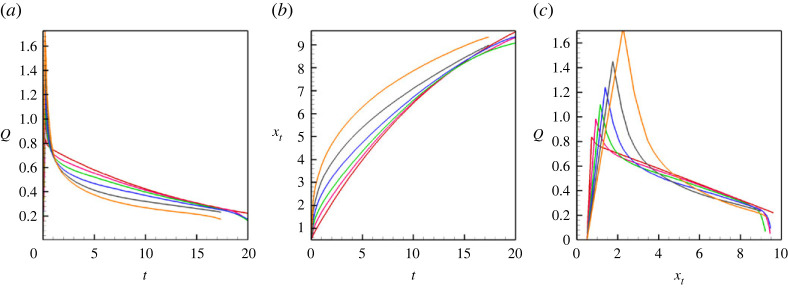
(*a*) The volumetric flow rate through the capillary versus time. (*b*) The position of the meniscus tip versus time. (*c*) The volumetric flow rate through the capillary versus the positions of the meniscus tip. The results are shown for a vertical capillary subjected to transversal vibrations. The calculations are fulfilled for the following parameters: A=−0.5, $Cn=10^{-4}$, Re=0.1, Pe=100, Gr=100, $M=10^{-5}$, and different values of the vibrational parameter V: V=0 (dashed line), V=8000 (red line), V=16 000 (green line), V=32 000 (blue line), V=64 000 (black line) and V=128 000 (orange line). (Online version in colour.)

Finally, figures 11 and 12 depict the flow fields and the integral characteristics for the displacement flow in a vertical capillary that is subjected to transversal vibrations. One can see that the effect of vibrations is very similar to the case of horizontal capillary. For a vertical capillary, there is an additional complexity due to the influence of the gravity force that makes the flow time (position) dependent (as shown in figure 12*a*,*c*). Nevertheless, one can easily see that vibrations deform the meniscus, stretching it along the capillary’s centreline. The vibrations slow down the flow, increasing the capillary pressure associated with the meniscus.

### (c) Longitudinal vibrations

**Figure 13.**
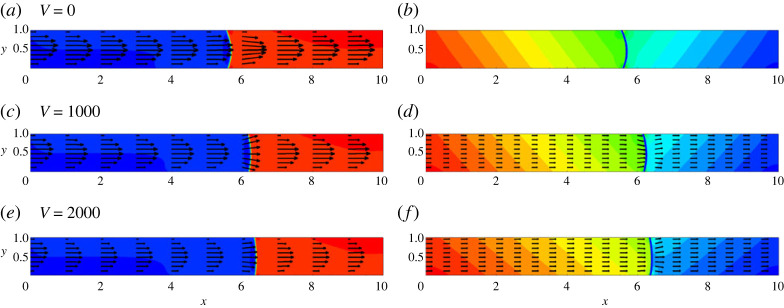
The field of concentration and velocity (*a*,*c*,*e*) and the fields of pressure and pulsation velocity (b,d,f) at t=10. The snapshots are shown for the horizontal capillary that is subjected to the longitudinal vibrations. The calculations are fulfilled for the following parameters: A=−0.5, $Cn=10^{-4}$, Re=0.1, Pe=100, Gr=100, $M=10^{-5}$ and three levels of vibrations, V=0 (*a*,*b*), V=1000 (*c*,*d*) and V=2000 (*e*,*f*). (Online version in colour.)

**Figure 14.**
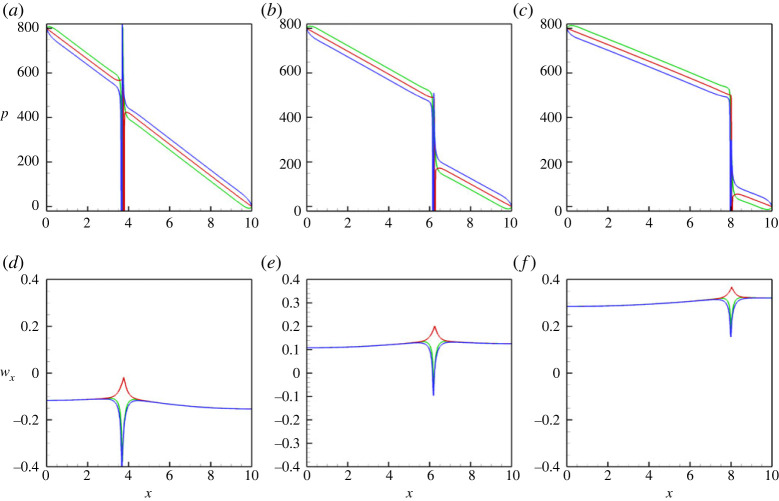
The profiles of pressure (*a*–*c*) and x-component of the vibrational velocity (*d*–*f*) along the capillary are shown for the three different positions: (green line) at the lower wall, y=0, (red line) at the centreline, y=1/2, and (blue line) at the upper wall, y=1. The profiles are shown for the horizontal capillary subjected to longitudinal vibrations at t=5 (*a*,*d*), t=10 (*b*,*e*) and t=15 (*c*,*f*). The calculations are fulfilled for the following parameters: A=−0.5, $Cn=10^{-4}$, Re=0.1, Pe=100, Gr=100, $M=10^{-5}$ and V=1000. (Online version in colour.)

**Figure 15.**
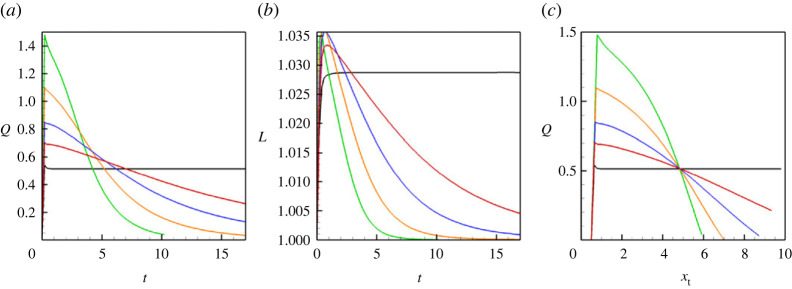
The volumetric flow rate through the capillary versus time (*a*), the length of the meniscus versus time (*b*) and the volumetric flow rate versus the position of the meniscus tip (*c*). The results are shown for the horizontal capillary subjected to transversal vibrations. The profiles are shown for the horizontal capillary subjected to longitudinal vibrations. The calculations are fulfilled for the following parameters: A=−0.5, $Cn=10^{-4}$, Re=0.1, Pe=100, Gr=100, $M=10^{-5}$ and different levels of vibrations, V=0 (black line), V=1000 (red line), V=2000 (blue line), V=4000 (orange line) and V=8000 (green line). (Online version in colour.)

Next, let us consider the case where the capillary is vibrated in the direction of its axis. Figure 13 depicts the typical shapes of the meniscus in a horizontal capillary that is subjected to the longitudinal vibrations. Figure 14 depicts the profiles of pressure and the profiles of x-component of the pulsational velocity at different moments. Figure 15*a*,*b* depicts the time variations of the flow rate and the time variations of the meniscus length. Figure 15*c* depicts the values of the flow rate as a function of the meniscus’s position in a capillary.

The figures indicate that the effect of longitudinal vibrations is stronger (if compared with the similar effects induced by transversal vibrations), namely, vibrations that are characterized by the vibrational parameter V>100 are already capable of visibly altering the displacement flow. Vibrations flatten menisci and alter the capillary pressure.

The principal action of the longitudinal vibrations is easy to explain. Indeed, if one considers two semi-infinite liquids separated by a flat boundary (the concentration field is the function of the coordinate that is perpendicular to the interface), and subjected to the vibrational forcing that is perpendicular to the interface, then one should say that for this configuration in the state of quasi-equilibrium (when there is no average flow), there is no pulsational velocity, and hence, the vibrational force (and the effect of vibrations) are non-existent. In the case of longitudinal vibrations, vibrations are perpendicular to the meniscus, and hence the action of vibrations does not exist when the meniscus is flat. Vibrations tend to flatten the meniscus (see figure 15*b*). However, in the displacement flow, the meniscus remains stretched by the imposed pressure difference. For a curved (stretched) meniscus, the concentration field is complex (the function of two coordinates), resulting in generation of the non-zero vibrational force, which affects the displacement dynamics.

In addition, we observe that the effect of vibrations depends on the position of the meniscus in a capillary. Vibrations assist the flow, until the meniscus reaches the middle point. When the meniscus is at the middle, the vibrations have no net effect on the average flow fields. When the meniscus passes the middle point, vibrations start to slow down the flow. Stronger vibrations can even counter-balance the action of an externally imposed pressure gradient, locking the fluids in a capillary.

One can also see that for the longitudinal vibrations, the values of the pulsational velocity (namely, the values of the x-component of the pulsational velocity) differ from zero along the entire length of the capillary: the fluid slips along the capillary with the fastest motion observed near the menisci. Moreover, when the meniscus passes through the middle point of the capillary, the x-component of the pulsational velocity changes its sign (see figure 14*d*,*e*,*f*), which results in the change of the sign of the vibrational effect. Also, one can notice that the oscillations of the meniscus tip and oscillations of the points of contact of the meniscus with the capillary walls do not happen in phase.

**Figure 16.**
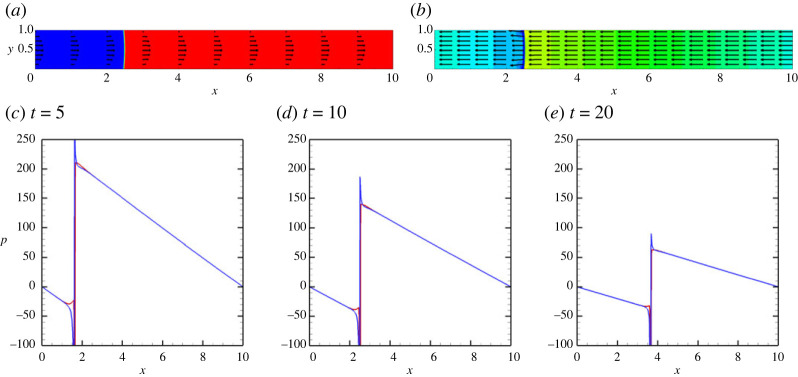
The field of concentration and velocity (*a*) and the fields of pressure and pulsation velocity (*b*) at t=10. (*c*,*d*,*e*) The pressure profiles along the capillary at three different time moments. The calculations are fulfilled for the horizontal capillary that is subjected to the longitudinal vibrations for the following parameters: A=−0.5, $Cn=10^{-4}$, Re=0.1, Pe=100, Gr=0, $M=10^{-5}$ and V=1000. For this calculation, there is no externally applied pressure difference between the ends of the capillary. (Online version in colour.)

As noted earlier, for the longitudinal vibrations, the action of vibrations competes with the action of the imposed pressure gradient. To elucidate the effect of vibrations, we also considered a capillary that is subjected to vibrations, but no pressure difference between the ends of a capillary is applied. Also, we assumed that there is no gravity. In this case, we still see that the meniscus is driven towards the middle point of a capillary, and when the meniscus is in the middle, the fluid system reaches the state of quasi-equilibrium, when no further (time-averaged) movements are observed. These results are illustrated in figure 16.

In this section, we do not show the results for the vertical capillaries that are subjected to longitudinal vibrations, as these results do not bring any new observations: still, the vibrations will flatten the meniscus and change the flow rates, and the action of vibrations will depend on the position of the meniscus in respect to the capillary’s middle point.

## 5. Conclusion

In this work, we assessed the action of the small-amplitude high-frequency vibrations on an immiscible liquid/liquid displacement flow through a capillary. We assumed that the capillary was initially saturated with one liquid, and the other liquid was injected from one of the capillary’s ends (and, respectively, the other liquid was displaced from the other end). The capillary was subjected to translational vibrations. We analyzed the action of the translational vibrations in two directions, along and across the capillary’s axis (longitudinal and transversal vibrations, respectively). Finally, we also assumed that the capillary was a part of a large porous matrix. The whole matrix was subjected to vibrations, and the fluids that saturated the matrix moved together with the solid parts of the matrix (in the first approximation). The time-averaged approach was applied to assess the action of the vibrations.

We found that vibrations (with intensity defined by the value of the vibrational parameter V) can alter the displacement flow. Vibrations alter the shape of a meniscus, turning the interface perpendicular to vibrations, and thus reshaping a classical piston-like meniscus into a sharp cone when the vibrations are directed across the capillary’s axis, and flattening a meniscus, when vibrations are directed along the capillary. The effect of vibrations depends on the value of the surface tension coefficient associated with interface: stronger vibrations are needed to alter the displacement flows with higher surface tension forces, and interfaces with lower surface tensions (miscible interfaces) could be controlled by a relatively weak vibration forcing.

Vibrations also alter the capillary pressures associated with interfaces. In the case of transversal vibrations, the capillary pressure is increased, and consequently, the flow is slowed down. In the case of longitudinal vibrations, the action of the vibrations depends on the position of the meniscus. Vibrations tend to bring the meniscus to the middle of the capillary, which is counteracted by the pressure gradient that is imposed between the capillary’s ends. For smaller levels of vibrations, vibrations assist the flow when a meniscus moves towards the middle point (the capillary pressure is decreased), and vibrations slow down the movement of the fluids when the meniscus passes the middle point (the capillary pressure is increased). For stronger vibrations, the effect imposed by vibrations can be so strong that fluids become locked inside the capillary (the capillary pressure becomes greater than an externally imposed pressure difference).

We should note that the changes of the capillary pressure are solely explained by reshaping of menisci by vibrations. Vibrations do not change the surface tension (distribution of fluids within a thin interface).

Also, vibrations induce pulsational movements of the fluids (to leading order the fluids oscillate together with the capillary, but at the next order of approximation, fluids will slip along the capillary). For translational vibrations, these pulsational movements are bounded to the area near a meniscus. For longitudinal vibrations, the pulsational movements extend to the entire capillary. The parabolic profile of the average flow remains unaffected by vibrations.

We should say that we cannot fully explain the dependence of the effect of the longitudinal vibrations on the position of the meniscus. The solution of the pulsational equations is determined by the concentration field and by the boundary conditions. We produced the simulations for different parameters, different gravity directions, and the capillaries of different lengths. We always observe the similar behaviour, which we need to attribute to the accepted geometry and boundary conditions. We need also to recall that we assume that the capillary is a building element of a larger porous matrix. The matrix will include vertical boundaries that will negate the pulsational flow in the horizontal direction. These boundaries are, however, not included in the geometry of our current work. Thus, to investigate this question further, we need to consider a more complex geometry that would include the other elements of a porous matrix, which we leave for the future work.

In conclusion, we should also note that in this work we examined a multiphase system with a single interface. The effect of vibrations could obviously be made stronger by taking a system with multiple interfaces, e.g. a droplet in a capillary or even a more complex liquid/liquid interface in a porous matrix. We would like also to note that there is a common expectation that shaking of a porous matrix facilitates the release of fluids that are entrapped in a matrix by capillary forces. We found that the effect of high-frequency vibrations on the movements of multiphase fluids in single capillaries (which are the building elements of matrices) is rather opposite: vibrations (or shaking) tend to either slow down or completely stop the displacement flows. However, a separate analysis fulfilled for the network of interconnected capillaries is needed to draw more reliable conclusions for porous media. Such an analysis is the next aim of our research work.

## Data Availability

This article has no additional data.

## Footnotes

1 Another option of inducing oscillatory motion in a porous medium is adding periodic modulation to the pressure difference that is imposed between the ends of the capillary. This would be a different problem statement, as the pressure pulsations will cause the fluid to slip along the matrix; we do not consider this effect.

2 We would like to note that for displacement flows the choice of the static contact angle is of little significance. The static contact angle defines the shape of a static meniscus. A displacement flow is, however, determined by the dynamic contact angle, whose value is independent of the static contact angle, and it is instead defined by the flow rate [13,14]. Thus, an accurate setting of the wetting properties would be a secondary issue for the current problem that is left for another study.

3 It is helpful to understand that the capillary joins two semi-infinite liquid domains. If there is no imposed pressure difference, then the state of equilibrium would correspond to the state when the boundary between the liquids is placed in the middle of the capillary. The initial position of the boundary is however different, and gravity tends to bring the boundary to its equilibrium position. This tendency explains our observations.

## References

[1] Beresnev IA, Johnson PA. 1994 Elastic-wave stimulation of oil production: a review of methods and results. Geophysics **59**, 1000-1017. (10.1190/1.1443645)

[2] Roberts PM, Venkitaraman A, Sharma MM. 1996 Ultrasonic removal of organic deposits and polymer induced formation damage. SPE Drill. Complet. **15**, 19-24. (10.2118/62046-PA)

[3] Aarts ACT, Ooms G. 1998 Net flow of compressible viscous liquids induced by travelling waves in porous media. J. Eng. Math. **34**, 435-450. (10.1023/A:1004314014329)

[4] Dezhkunov NV, Leighton TG. 2004 Study into correlation between the ultrasonic capillary effect and sonoluminescence. J. Eng. Phys. Thermophys. **77**, 53-61. (10.1023/B:JOEP.0000020719.33924.aa)

[5] Pride SR, Flekkoy EG, Aursjo O. 2008 Seismic stimulation for enhanced oil recovery. Geophysics **73**, O23-O35. (10.1190/1.2968090)

[6] Kuznetsov OL, Simkin EM, Chilingar GV, Gorfunkel MV, Robertson JO. 2002 Seismic techniques of enhanced oil recovery: experimental and field results. Energy Sources **24**, 877-889. (10.1080/00908310290086761)

[7] Lyubimov DV, Cherepanov AA, Lyubimova TP, Roux B. 1997 Interface orienting by vibration. C. R. Acad. Sci. Paris **325**, 391-396. (10.1016/S1251-8069(97)80068-1)

[8] Lyubimov DV, Lyubimova TP, Cherepanov AA. 2003 Dynamics of interfaces subject to vibrations. Moscow, Russia: FizMatLit.

[9] Vorobev A, Lyubimova T. 2019 Vibrational convection in a heterogeneous binary mixture. Part I. Time-averaged equations. J. Fluid Mech. **870**, 543-562. (10.1017/jfm.2019.282)

[10] Vorobev A, Lyubimova T. 2019 Vibrational convection in a heterogeneous binary mixture. Part II. Frozen waves. J. Fluid Mech. **870**, 543-562. (10.1017/jfm.2019.282)

[11] Vorobev A. 2010 Boussinesq approximation of the Cahn-Hilliard-Navier-Stokes equations. Phys. Rev. E **82**, 056312. (10.1103/PhysRevE.82.056312) 21230581

[12] Vorobev A, Prokopev S, Lyubimova T. 2020 Phase-field modelling of a liquid/liquid immiscible displacement through a network of capillaries. J. Comput. Phys **421**, 109747. (10.1016/j.jcp.2020.109747)

[13] Prokopev S, Vorobev A, Lyubimova T. 2019 Phase-field modeling of an immiscible liquid-liquid displacement in a capillary. Phys. Rev. E **99**, 03313. (10.1103/PhysRevE.99.033113) 30999476

[14] Ngan CG, Dussan EB. 1982 On the nature of the dynamic contact angle: an experimental study. J. Fluid Mech. **118**, 27-40. (10.1017/S0022112082000949)

[15] Vorobev A, Prokopev S, Lyubimova T. 2021 Non-equilibrium capillary pressure of a miscible meniscus. Langmuir **37**, 4817-4826. (10.1021/acs.langmuir.0c03633) 33856813

[16] Lyubimova T, Vorobev A, Prokopev S. 2019 Rayleigh-Taylor instability of a miscible interface in a confined domain. Phys. Fluids **31**, 014104. (10.1063/1.5064547)

